# Predicting protein-protein interactions in unbalanced data using the primary structure of proteins

**DOI:** 10.1186/1471-2105-11-167

**Published:** 2010-04-02

**Authors:** Chi-Yuan Yu, Lih-Ching Chou, Darby Tien-Hao Chang

**Affiliations:** 1 Graduate Institute of Biomedical Electronics and Bioinformatics, National Taiwan University, Taipei 106, Taiwan; 2 Department of Electrical Engineering, National Cheng Kung University, Tainan 70101, Taiwan

## Abstract

**Background:**

Elucidating protein-protein interactions (PPIs) is essential to constructing protein interaction networks and facilitating our understanding of the general principles of biological systems. Previous studies have revealed that interacting protein pairs can be predicted by their primary structure. Most of these approaches have achieved satisfactory performance on datasets comprising equal number of interacting and non-interacting protein pairs. However, this ratio is highly unbalanced in nature, and these techniques have not been comprehensively evaluated with respect to the effect of the large number of non-interacting pairs in realistic datasets. Moreover, since highly unbalanced distributions usually lead to large datasets, more efficient predictors are desired when handling such challenging tasks.

**Results:**

This study presents a method for PPI prediction based only on sequence information, which contributes in three aspects. First, we propose a probability-based mechanism for transforming protein sequences into feature vectors. Second, the proposed predictor is designed with an efficient classification algorithm, where the efficiency is essential for handling highly unbalanced datasets. Third, the proposed PPI predictor is assessed with several unbalanced datasets with different positive-to-negative ratios (from 1:1 to 1:15). This analysis provides solid evidence that the degree of dataset imbalance is important to PPI predictors.

**Conclusions:**

Dealing with data imbalance is a key issue in PPI prediction since there are far fewer interacting protein pairs than non-interacting ones. This article provides a comprehensive study on this issue and develops a practical tool that achieves both good prediction performance and efficiency using only protein sequence information.

## Background

Various interactions among proteins are essential to diverse biological functions in a living cell. Information about these interactions provides a basis to construct protein interaction networks and improves our understanding of the general principles of the workings of biological systems [1]. The study of protein-protein interaction (PPI) is, therefore, an important theme of systems biology [2]. Recent years have seen the development of experimental approaches to analyze PPIs, including yeast two-hybrid (Y2H) [3,4], coimmunoprecipitation (CoIP) [5-7] and other approaches [8,9]. The resulting interaction data is publicly available in several databases such as BIND [10], DIP [11], MIPS [12] and IntAct [13].

While experimentally detected interactions present only a small fraction of the real PPI network [14,15], many computational methods have been developed to provide complementary information for experimental approaches. Some of these computational methods require not only sequence information but also auxiliary data, for example, localization data [16], structural data [17-19], expression data [20,21] and/or interactions from orthologs [22,23]. Shoemaker and Panchenko have provided a comprehensive review of these computational methods [24].

The main limitation of above methods is that they rely on prior knowledge that may be expensive to acquire. To overcome this limitation, several *de novo *(*ab initio*) algorithms have been proposed to detect potential interacting proteins for which no auxiliary information are available [25-34]. Most of these *de novo *PPI predictors transform protein sequences into feature vectors and adopt supervised machine learning (ML) techniques to analyze these feature vectors. Najafabadi and Salavati proposed a method based on codon usage [35], which utilizes DNA sequence for feature extraction and requires open reading frame (ORF) information. The adopted ML techniques include random decision forests [28] and support vector machines (SVMs) [25,27,29,33,34]. These ML-based approaches achieved satisfactory performance on the input datasets comprising equal number of interacting and non-interacting protein pairs. However, this ratio is not balanced in nature, and these methods were not comprehensively evaluated with respect to the effect of the large number of non-interacting pairs in a naturally unbalanced dataset [36,37]. This unbalanced characteristic of PPI datasets, as will be elaborated in this study, requires more attention when developing and evaluating PPI prediction methods.

This study presents a novel ML-based method using only the primary sequences to predict interacting proteins. The proposed feature set is improved from the conjoint triad feature [33], which describes a protein sequence by the frequencies of distinct conjoint triads--three continuous amino acids--observed in it. We propose a probability-based mechanism for estimating the significance of triad occurrences considering the amino acid composition. This improvement is designed to mitigate the dependency of triad occurrence frequencies on the amino acid distribution. Another notable contribution of this study is to provide a systematic analysis of the effect of dataset sampling when evaluating prediction performance.

This article reports several experiments conducted to evaluate the present *de novo *PPI predictor. A large collection of 17,855 interacting pairs from 6,429 proteins are adopted to generate different unbalanced datasets with 1:1~1:15 positive-to-negative ratios. As illustrated by the experimental results, the proposed feature set achieves the best prediction performance when compared with two *de novo *feature sets recently published for PPI prediction. Furthermore, the analyses included in this study reveal that a) the extent of imbalance of the sampled dataset and b) the efficiency of the employed classification algorithm are important to PPI predictors.

## Results and Discussion

In this section, a quick overview of the proposed method is first presented, where the details are left in the Methods section. The issues of handling unbalanced data are then addressed to reveal the importance of data imbalance in experiments, followed by experimental results. The end of this section discusses some considerations for real world PPI data.

### Proposed PPI predictor

The present method uses only sequence information for training a classifier to distinguish positive instances of truly interacting protein pairs from negative instances of non-interacting protein pairs. Shen *et al. *[33] have shown that the triad frequency is a useful feature encoding for PPI prediction. However, the frequencies of different triads are largely affected by the amino acids distribution. Thus, a probability-based mechanism of estimating triad significance is proposed to alleviate the effect of the amino acid distribution in nature. The relaxed variable kernel density estimator (RVKDE), recently proposed by the authors [38], is then invoked to predict protein-protein interactions based on the feature vectors. The details of how to transform protein pairs into feature vectors, the algorithm of RVKDE classifier and some implementation issues can be found in the Methods section.

### Noteworthy issues for predicting unbalanced datasets

**Figure 1:**
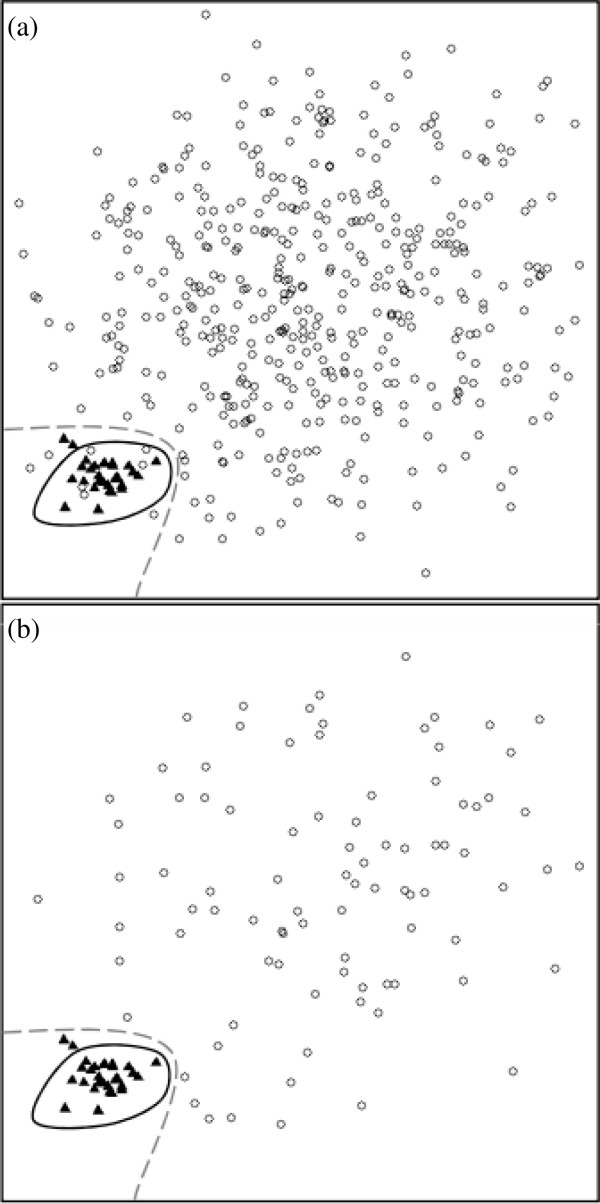
**Demonstration of evaluation bias owing to subset-sampled dataset, where the dashed line represents the decision boundary of *Predictor*_*A *_while the solid line represents the decision boundary of *Predictor*_*B*_**.

In PPI prediction, positive instances are collected from PPI databases, while negative instances are all other protein pairs. This is a large and extremely unbalanced data. A common practice in processing such unbalanced datasets is to form a balanced dataset by sampling from the original dataset. This step of sampling, however, raises new problems. For example, Figure 1(a) shows a synthesized 1:15 dataset (where the positive-to-negative ratio is 1:15). Figure 1(b), which is a sampled 1:3 dataset, contains all the positive instances and arbitrarily selected negative instances from Figure 1(a). *Predictor*_*A *_and *Predictor*_*B*_--represented by their decision boundaries--are two predictors. Both predictors perform better on the sampled dataset than the original dataset. After sampling, some of negative instances that are close to the positive cluster are excluded. That leads to an easier dataset for classification. Another observation from Figure 1 relates to the performance differences between the two predictors. *Predictor*_*B *_is obviously better than *Predictor*_*A *_in Figure 1(a). However, in a sampled data such as Figure 1(b), *Predictor*_*B *_looks to have comparable performance with *Predictor*_*A*_. As a result, sampling to create balanced datasets not only simplifies the problem, but also prevents a realistic comparison of different predictors.

This study uses unbalanced datasets of different positive-to-negative ratios for performance evaluation to elucidate how the sampled datasets affect the prediction performance. However, handling unbalanced datasets leads to two problems. The first problem is choosing a suitable evaluation measure. Table 1 shows five widely used measurements for binary classification problems. A predictor which simply predicts all samples as negative will have an *accuracy *of 93.8% in a 1:15 dataset. Despite the appealing score, this strategy is useless because it cannot predict any potential interactions. Maximizing *accuracy *leads predictors to favor the majority group. In PPI prediction, however, we care more about the interacting pairs, which are the minority group. *F-measure *is a more appropriate measurement because it is the harmonic mean of *precision *and *sensitivity*, both of which are related to the performance of the positive instances [39].

**Table 1:** Evaluation measurements employed in this study

Measurement	Abbreviation	Equation^1^
*Precision*	*Prec.*	TP/(TP+FP)
*Sensitivity *(*recall*)	*Sens.*	TP/(TP+FN)
*Specificity*	*Spec.*	TN/(TN+FP)
*Accuracy*	*Acc.*	(TP+TN)/(TP+TN+FP+FN)
*F-measure*	*Fm.*	2TP/(2TP+FP+FN)

^1^The definition of the abbreviations used: TP is the number of interacting protein pairs correctly classified; FN is the number of interacting protein pairs incorrectly classified as non-interacting; TN is the number of non-interacting protein pairs correctly classified; and FP is the number of non-interacting protein pairs incorrectly classified as interacting.

Another problem of processing unbalanced datasets is the increasing size. For example, using the widely used LIBSVM package [40] to analyze a 1:1 dataset containing 33,710 protein pairs requires 14,059 seconds. The execution time is measured on a workstation equipped with an Intel Core 2 Duo E8400 3.0 GHz CPU and 8 GB memory, and do not include the time taken to carry out parameter selection or cross validation. According to the observed time complexity of SVM [41], a complete parameter selection on a 1:15 dataset of the same amount of positive samples may take months or even years using a contemporary workstation. Conversely, analyzing the same 1:1 dataset with RVKDE takes only 142 seconds on the same workstation mentioned above, allowing for the analysis of unbalanced datasets within a reasonable time. On the other hand, replacing SVM with RVKDE sacrifices a slight prediction performance of 0.3% *F-measure *(from 80.7% to 80.4%) on a 1:1 dataset used in this study. Thus, this study employs RVKDE as the classification algorithm to compare alternative feature sets. Its efficiency is essential for handling highly unbalanced datasets.

### Evaluation of the proposed feature set

#### Datasets

This study adopts a collection of protein-protein interactions from the Human Protein Reference Database (HPRD) [42,43], Release 7. This version of HPRD contains 38,167 PPI and 25,661 protein entries from literature. Interactions in which more than two proteins participate are removed, since it is difficult to confirm which individual proteins in such complexes have physical interactions [21]. Furthermore, interactions that contain a protein sequence with selenocysteine (U) are also removed. The remaining set comprises 37,044 interacting protein pairs in which 9,441 proteins are involved. Since interactions detected based on *in vitro *experiments might be false positives that occur in laboratory procedures but do not occur physiologically [14], only the *in vivo *PPI pairs are used in preparing the positive set to prevent introducing spurious interactions. The resultant positive set comprises 17,855 interacting protein pairs and 6,429 proteins. This study follows the procedure in a previous work [33] to construct a negative set, which ensures that (a) a negative sample is not in any of the 38,167 interactions (including *in vitro *and those with >2 participated proteins) and (b) the two individual proteins of a negative instance are included in the 6,429 proteins of the positive set. Thus, a dataset of *m *interacting pairs that contain *n *proteins can generate *n*(*n *+ 1)/2 - *m *negative instances.

This work arbitrarily divides the 17,855 positive instances into two subsets for the training and testing sets, respectively. The training set includes 16,855 positive instances and the testing set includes the remaining 1,000 positive instances. Datasets with different positive-to-negative ratios are generated with the same positive instances and distinct negative sets, which are obtained by randomly sampling from the negative instances. Care has been taken to ensure that a negative instance would not be selected in more than one set. Since the procedure to generate training and testing datasets involves randomness, the prediction process is repeated 20 times to alleviate the evaluation bias in a single prediction process. All the training and testing datasets with different positive-to-negative ratios are available at http://mbi.ee.ncku.edu.tw/ppi/ppi.tgz, for any following studies of PPI prediction that require unbalanced datasets as a benchmark to compare with.

### Comparison with similar works

This study adopts a large collection of protein-protein interactions to illustrate the importance of dataset imbalance. Hence, the present significance vector is compared with two advanced feature sets recently published for *de novo *PPI prediction that have been shown delivering good performance on large datasets (>5,000 PPIs) [33,34]. The first feature set was developed by Shen *et al.*, which employed the frequency of conjoint triads as the feature set [33]. This feature set has been reported to achieve >82.23% *precision*, >84.00% *sensitivity *and >82.75% *accuracy*, and is the first study of conducting large-scale experiments on the whole HPRD data to show its robustness and reliability. The second feature set was developed by Guo *et al.*, which proposed a feature representation using auto cross covariance [34]. This feature set has a reported *accuracy *of 87.36% on the PPI data of yeast *Saccharomyces cerevisiae*, and also achieved an *accuracy *of 88.09% on another independent data set of yeast PPIs.

**Figure 2:**
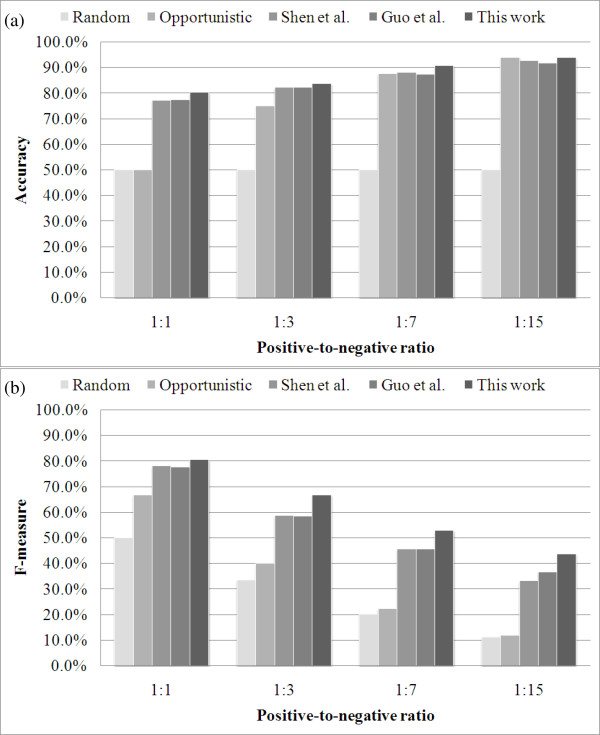
**Comparison of *accuracy *and *F-measure *in datasets with different positive-to-negative ratios**. The Random predictor predicts any query protein pair as positive with a probability of 0.5, and as negative with a probability of 0.5, too. The Opportunistic predictor predicts any query protein pair as negative for *accuracy *and it predicts any query protein pair as positive for *F-measure*. Shen *et al. *use triad frequency as the feature set. Guo *et al. *use auto cross covariance as the feature set. This work uses triad significance as the feature set.

Table 2 shows the prediction performances using different features sets. In Table 2, the proposed feature set achieves the best performance in most positive-to-negative ratios and evaluation measurements. To further investigate the effects of data imbalance, Figure 2 extracts *accuracy *and *F-measure *from Table 2, and introduces two trivial predictors as baseline candidates. The random predictor predicts any query protein pair as positive with a 0.5 probability, and as negative with a 0.5 probability as well. The opportunistic predictor learns nothing form the training set but can ingratiate its prediction strategy with alternative measurements: (a) it predicts any query protein pair as negative for *accuracy *and (b) it predicts any query protein pair as positive for *F-measure*.

**Table 2:** Performance of the compared feature sets on datasets with different positive-to-negative ratios

Feature	*Acc*. (%)	*Fm*.^1 ^(%)	*Prec*. (%)	*Sens*. (%)	*Spec*. (%)
Datasets with 1:1 positive-to-negative ratio					
Shen *et al.*^2^	77.1 ± 0.8	77.9 ± 0.8	75.2 ± 0.9	80.9 ± 1.4	73.3 ± 1.4
Guo *et al.*^3^	77.2 ± 0.9	77.6 ± 0.9	76.2 ± 1.0	79.1 ± 1.3	75.4 ± 1.4
This work^4^	**80.1 ± 0.8**	**80.4 ± 0.8**	**79.4 ± 1.0**	**81.4 ± 1.4**	**78.8 ± 1.4**
Datasets with 1:3 positive-to-negative ratio					
Shen *et al.*	82.2 ± 0.3	58.6 ± 1.1	**69.9 ± 0.8**	50.4 ± 1.6	92.7 ± 0.3
Guo *et al.*	82.1 ± 0.6	58.3 ± 1.7	69.8 ± 1.6	50.1 ± 1.8	**92.8 ± 0.4**
This work	**83.6 ± 0.5**	**66.7 ± 1.2**	67.9 ± 0.9	**65.5 ± 1.7**	89.7 ± 0.4
Datasets with 1:7 positive-to-negative ratio					
Shen *et al.*	88.0 ± 0.3	45.4 ± 1.7	52.8 ± 1.8	39.9 ± 1.9	94.9 ± 0.3
Guo *et al.*	87.2 ± 0.3	45.5 ± 1.3	48.8 ± 1.5	**42.6 ± 1.3**	93.6 ± 0.3
This work	**90.6 ± 0.2**	**52.8 ± 1.7**	**71.5 ± 1.5**	41.8 ± 1.8	**97.6 ± 0.2**
Datasets with 1:15 positive-to-negative ratio					
Shen *et al.*	92.5 ± 0.1	33.1 ± 1.4	37.5 ± 1.3	29.7 ± 1.5	96.7 ± 0.1
Guo *et al.*	91.7 ± 0.2	36.6 ± 1.5	35.1 ± 1.5	38.3 ± 1.9	95.3 ± 0.2
This work	**93.7 ± 0.2**	**43.6 ± 1.3**	**49.5 ± 1.7**	**39.0 ± 1.3**	**97.3 ± 0.1**

The best performance among each positive-to-negative ratio is highlighted with bold font. ^1^The parameter selection is based on a five-fold cross validation of the training dataset to maximize the *F-measure*. ^2^Using triad frequency as the feature set. ^3^Using auto cross covariance as the feature set. ^4^Using triad significance as the feature set.

**Figure 3:**
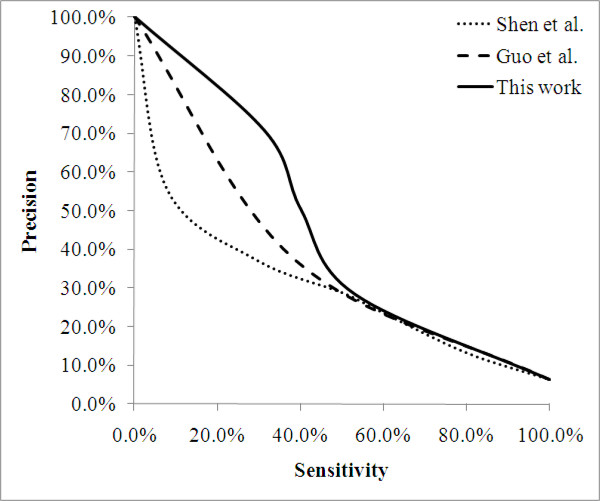
***Precision *vs. *sensitivity *curve for the dataset of 1:15 positive-to-negative ratio**.

In Figure 2, the present feature set has an advantage of ~3% *accuracy *on the 1:1 dataset, but this advantage decreases as the positive-to-negative ratio is getting more unbalanced. The advantage is only 1.2% and 2.0% on the 1:15 dataset. Conversely, this feature set has an advantage of <3% *F-measure *on the 1:1 dataset. This advantage increases with the positive-to-negative ratio, leading to an advantage of 10.5% and 7.0% on the 1:15 dataset. The different trends between the two measurements are reasonable and could be explained by the performance of the opportunistic predictor. For *accuracy*, the opportunistic predicts all query protein pairs as negative, thus a high accuracy can be achieved in an extremely unbalanced dataset without detecting any interacting pairs. On the other hand, both trivial predictors deliver decreasing performances as the dataset gets more unbalanced in terms of *F-measure*. These results imply that the problem is getting harder as the dataset is getting more unbalanced, which concurs with the observations elaborated in Figure 1. Figure 3 shows the *precision *vs. *sensitivity *curve for the 1:15 dataset, where the proposed significance vector generally performs better than the two compared works when precision is greater than 30%.

### Considerations for real word data

We have presented a predictor that is consistently better than the compared methods on datasets of varying data imbalance, but a critical question is how the predictor would perform on real world data. This is an open question for not only PPI prediction but many other bioinformatics fields, and there are currently no satisfactory solutions.

To illustrate this issue, we create a dataset of all the protein pairs from the 6,429 proteins present in the HPRD *in vivo *PPI dataset. This dataset contains 17,855 *in vivo *PPIs and 20,631,068 negative samples that are not in any of the 38,167 HPRD interactions, and has an extreme positive-to-negative ratio of ~1:1100, which we will call the comprehensive dataset. Training with such a comprehensive dataset would take more than 400 days using RVKDE and decades using SVM. Hence, we use a lower ratio (1:7) for training then test the model on a dataset with the desired positive-to-negative ratio. Such a comprehensive dataset, however, would likely contain many false negative samples (*i.e.*, interacting protein pairs in the negative dataset) given the incompleteness of the human protein-protein interaction data, biasing the results. Some previous studies proposed to restrict negative samples that are located in different cellular compartments to avoid such false negative samples [34,35]. A second dataset is thus created by removing the negative samples of which the two proteins are in the same cellular compartment from the comprehensive dataset. This dataset, which we call the compartmental dataset, contains 975,626 negative samples and has a positive-to-negative ratio of ~1:55.

The *F-measure *of the present method on the comprehensive dataset is 2.93%. This performance is overly pessimistic due to the incompleteness of human protein interactions network (PIN). Based on current understanding, the size of PIN comprises ~650,000 interactions [44]. However, less than 3% interactions are currently identified and collected in HPRD. Namely, even a perfect predictor cannot deliver an *F-measure *greater than 6%. On the other hand, the *F-measure *on the compartmental dataset achieves 57.4%. Note that this *F-measure *is higher than those obtained by evaluating on the 1:7 and 1:15 datasets in Table 2. The process of removing false negatives also removes the true negatives localized in the same cellular compartment that are difficult to discriminate from interacting pairs, making the problem easier. It might reduce the learning problem to that of classifying whether two proteins are in the same cellular compartment.

In summary, the realistic performance drops in between 2.93% and 57.4%. This wide range reveals a) the impact on the performance from different strategies of negative dataset construction and b) the difficulty in estimating the performance for real world data. Currently, *de novo *approaches are suitable to analyze a certain type of interactions (such as combinatorial interaction of transcription factors [45] or small molecule-kinase interactions [46]) that features a lower degree of imbalance, while more effort is needed to alleviate the decreasing performance from the degree of imbalance for general protein interactions. More work on evaluation schemes is also required to provide a reasonable and realistic test to assess PPI predictors.

## Conclusions

This article presents a novel method for predicting protein-protein interactions only using the primary sequences of proteins, which consistently outperforms other algorithms in the same category for a collection of datasets. We have used RVKDE, an efficient machine learning algorithm, to achieve an extensive evaluation on alternative approaches with highly unbalanced data. The results reveal the importance of dataset construction and the issue of data sampling in problems with naturally unbalanced distributions. Finally, a discussion on real world data is included, which show that much improvement in *de novo *PPI predictors are needed before they can be effectively used on general protein interactions.

## Methods

### Feature encoding

This work encodes each protein sequence as a feature vector by considering the amino acid triads observed in it. An amino acid triad regards three continuous residues as a unit. Each PPI pair is then encoded by concatenating the two feature vectors of the two individual proteins. However, considering all 20^3 ^amino acid triads requires a 16000-dimensional feature vector to encode a protein pair, which is too large for most practical classifiers, the 20 amino acid types are clustered into seven groups based on their dipole strength and side chain volumes to reduce the dimensions of the feature vector [33]. The seven amino acid groups are listed in Table 3.

**Table 3:** Amino acid groups adopted in this study

Group no.	Amino acids	Occurrence (%)^1^
1	Ala, Gly, Val	22.0
2	Ile, Leu, Phe, Pro	24.2
3	Tyr, Met, Thr, Ser	17.3
4	His, Asn, Gln, Tpr	11.4
5	Arg, Lys	11.4
6	Asp, Glu	12.2
7	Cys	1.4

This table follows the Shen et al.'s work [33]. ^1^Occurrences of seven amino acid groups in the Swiss-Prot database release 57.0 [49].

**Figure 4:**
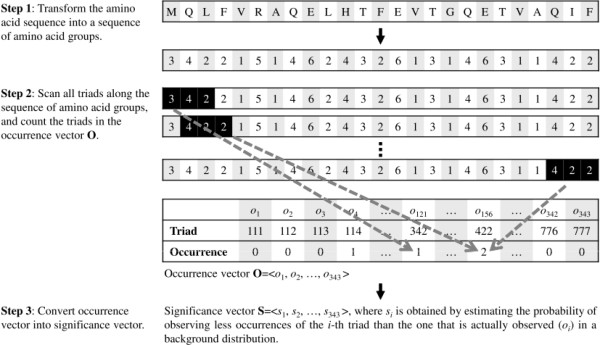
**Schematic diagram of encoding a protein sequence into a feature vector**.

The process of encoding a protein sequence is shown in Figure 4. First, the protein sequence is transformed into a sequence of amino acid groups. This method then scans triads one by one along the sequence of amino acid groups. Each scanned triad is counted in an occurrence vector, **O**, in which each element *o*_*i *_represents the number of the *i*-th type of triad observed in the sequence of amino acids groups. However, the value of *o*_*i *_might be highly correlated to the distribution of amino acids, *i.e.*, triads that consist of amino acid groups frequently observed in nature (*e.g.*, group 1 and 2) tend to have a large value of *o*_*i*_.

To solve this problem, this study proposes a significance vector, **S**, to replace the occurrence vector for representing a protein sequence. Here the significance of a triad is defined by answering the following question:

How rare is the number of observed occurrences considering the amino acid composition of the protein?

This definition, for example, distinguishes the significance of an occurrence of triad '111' appearing in two sequences that have equal length but contains three and ten group-1 residues. In this example, the occurrence of '111' in the former sequence is more significant since it is less likely to occur by chance. Accordingly, each element *s*_*i *_in the significance vector is formulated as follows:(1)

where *X*_*i *_is a random variable representing the number of observations of the *i*-th triad in a background distribution of protein sequences and Pr means the probability. We define *s*_*i *_as the probability of observing less occurrences of the *i*-th triad than the one that is actually observed (*o*_*i*_), which equals to 1 minus the p-value [47]. A common practice to estimate *X*_*i *_is to permute the original protein sequence many times while preserving its amino acid composition. Suppose that *x*_*ij *_is the number of the *i*-th triad observed in the *j*-th sequence from *n *permuted sequences, Eq. (1) can be re-formulated as(2)

In our current implementation, *n *is set to 10,000 to make each estimated *s*_*i *_vary less than 1% relative to the absolute value of *s*_*i*_. Accordingly, each protein sequence is represented as a significance feature vector, in which each element *s*_*i *_is calculated from *o*_*i *_with Eq. (2). For a protein pair, the two vectors of both protein sequences are concatenated to form a 686-dimensional feature vector.

### Relaxed variable kernel density estimator

This study adopts the RVKDE algorithm for accommodating to the large amount of negative instances in unbalanced datasets. One main distinctive feature of RVKDE is that it features an average time complexity of *O*(*n*log*n*) for carrying out the training process, where *n *is the number of instances in the training set. A kernel density estimator is in fact an approximate probability density function. Let {**s**_1_, **s**_2 _...**s**_*n*_} be a set of instances randomly and independently taken from the distribution governed by *f*_*X *_in the *m*-dimensional vector space. Then, with the RVKDE algorithm, the value of *f*_*X *_at point **v **is estimated as follows:

where

1. ;

2. *R*(**s**_*i*_) is the maximum distance between **s**_*i *_and its *ks *nearest training samples;

3. Γ (·) is the Gamma function [48];

4. *α*, *β *and *ks *are parameters to be set either through cross validation or by the user.

When using RVKDE to predict protein-protein interactions, two kernel density estimators are constructed to approximate the distributions of interacting and non-interacting protein pairs in the training set. A query protein pair (represented as the feature vector **v**) is predicted to the class that gives the maximum value among the two likelihood functions defined as follows:

where | *S*_*j *_| is the number of class-*j *training instances, and (**v**) is the kernel density estimator corresponding to class-*j *training samples. In this study, *j *is either 'interacting' or 'non-interacting'. Current RVKDE implementation includes only a limited number, denoted by *kt*, of nearest training samples of **v **to compute  (**v**) in order to improve the efficiency of the predictor. The parameter *kt *is set either through cross-validation or by the user.

As with SVM and other multivariate statistical learning tools, the performance of RVKDE depends on the parameter selection. The four parameters in RVKDE (*α*, *β*, *ks *and *kt*, see the Methods section for further information) are selected using a grid search approach to maximize *F-measure *of a five-fold cross validation on the training set.

## Competing interests

The authors declare that they have no competing interests.

## Authors' contributions

Author CYY designed the experiments and performed all calculations and analyses. LCC aided in interpretation of the data and manuscript preparation. DTHC designed the methodology and conceived of this study. All authors have read and approved this manuscript.
